# Enteropathogenic and Multidrug-Resistant *bla*_CTX-M_-Carrying *E. coli* Isolates from Dogs and Cats

**DOI:** 10.3390/ani14172463

**Published:** 2024-08-24

**Authors:** Catherine Biondo Feitosa, Gabriel Siqueira dos Santos, Natalia Carrillo Gaeta, Gustavo da Silva Schiavi, Carla Gasparotto Chande Vasconcelos, Jonas Moraes Filho, Marcos Bryan Heinemann, Adriana Cortez

**Affiliations:** 1 Curso de Pós-Graduação em Saúde Única, Universidade Santo Amaro, São Paulo 04743-030, Brazilnatalia.gaeta@usp.br (N.C.G.);; 2 Laboratório de Zoonoses Bacterianas, Departamento de Medicina Veterinária Preventiva e Saúde Animal, Faculdade de Medicina Veterinária e Zootecnia, Universidade de São Paulo, São Paulo 05508-220, Brazil; 3 Pós Graduação em Clínica Veterinária, Faculdade de Medicina Veterinária e Zootecnia da Universidade de São Paulo, São Paulo 05508-220, Brazil; 4 Laboratório Veterinário VidaVet, São Paulo 18602-060, Brazil

**Keywords:** EPEC, multidrug-resistant, ESBL, microbiota

## Abstract

**Simple Summary:**

Dogs and cats can carry enteropathogenic *Escherichia coli* (EPEC), and resistance to antimicrobials can impair eventual treatment. Ninety-seven isolates were collected from fecal samples of 31 dogs and 3 cats from Botucatu, Brazil, between March and October 2021. Twenty *E. coli* strains were identified as EPEC (20.6%), 5.1% had extended-spectrum β-lactamase (ESBL) production, and 13.4% were multidrug-resistant. Phylogroups A and B2 were predominant, comprising 29.9% and 26.8% of isolates, respectively. This study shows the prevalence of EPEC and antimicrobial-resistant *E. coli* strains in dogs and cats.

**Abstract:**

Enteropathogenic *Escherichia coli* (EPEC) are pathogens associated with gastrointestinal illnesses. Dogs and cats can harbor EPEC, and antimicrobial resistance may impair necessary treatments. This study characterized *E. coli* strains from dogs and cats, focusing on phylogroup classification, virulence factors, and antimicrobial resistance profiles. Ninety-seven *E. coli* isolates from fecal samples of 31 dogs and 3 cats were obtained from a private diagnostic laboratory in Botucatu, Brazil, from March to October 2021. The antimicrobial susceptibility was assessed using the disk diffusion method. Polymerase chain reaction (PCR) was employed to screen for *bla*_CTX-M_ and genes encoding virulence factors, as well as to classify the isolates into phylogroups. Twenty isolates were positive for intimin encoding gene *eae* and, consequently, these isolates were classified as EPEC (20.62%). Notably, 5.1% (5/97) of the isolates exhibited extended-spectrum β-lactamase (ESBL) production and 13.4% (13/97) were identified as multidrug-resistant bacteria. Phylogroups A and B2 were the most prevalent, comprising 29.9% (29/97) and 26.8% (26/97) of the bacterial isolates, respectively. This characterization highlights the prevalence of EPEC in domestic animals, emphasizing the potential risk they pose to public health and highlighting the urgency of responsible antimicrobial use in veterinary practices and the important role of laboratories in the surveillance of pathogenic multidrug-resistant bacteria.

## 1. Introduction

Diarrheagenic *Escherichia coli* (DEC) strains comprise a diverse group of bacteria frequently implicated in enteric infections. These strains can be categorized as pathotypes, based on their virulence factors, such as enteropathogenic *E. coli* (EPEC), enterotoxigenic *E. coli* (ETEC), enteroaggregative *E. coli* (EAEC), enteroinvasive *E. coli* (EIEC), Shiga toxin-producing *E. coli* (STEC), and enterohaemorrhagic *E. coli* (EHEC) [1].

The classification of EPEC strains is based on the production of intimin Eae, a protein responsible for bacterial attachment to gut epithelial cells. In addition to the production of Eae, if EPEC produces bundle-forming pili (BFP), it is classified as typical EPEC; if it does not, it is classified as atypical EPEC. Still, STEC strains produce Shiga-like toxins, specifically Stx1 and/or Stx2, and those expressing Eae and Stx1 and/or Stx2 fall into the EHEC pathotype [2]. There is evidence that adult domestic ruminants, mainly bovine, are natural reservoirs of EPEC, EHEC, and STEC strains [3,4]. This is not observed for calves, which are frequently reported to experience diarrhea due to the effect of DEC virulence factors [5].

There are reports of acute gastroenteritis associated with EPEC in dogs [6], but the impact of EPEC, STEC, and EHEC on the health of dogs and cats remains unclear. Even asymptomatic animals can serve as reservoirs for these pathotypes, potentially participating in the zoonotic transmission of DEC [7,8].

Apart from virulence factors, acquired antimicrobial resistance (AMR) in bacteria can pose a public health concern in cases of EPEC infections [8,9]. The proliferation of antimicrobial-resistant bacteria is a consequence of the widespread and inappropriate use of antimicrobials in both human and animal healthcare. This trend poses a substantial threat to public health as it undermines the efficacy of antimicrobial treatments, including cephalosporins, rendering them ineffective. Dogs and cats can also harbor multidrug-resistant (MDR) bacteria, and the zoonotic transmission of MDR and extended-spectrum beta-lactamase (ESBL)-producing *E. coli* may occur [8,9]. Likewise, humans can also transmit resistant bacteria to their pets [10].

Private laboratories frequently conduct bacterial culture, isolation, and antimicrobial susceptibility tests. Given their high volume of sample processing, these facilities possess significant potential to contribute to an extensive surveillance network aimed at identifying potential public health pathogens. However, they seldom fulfill this role in practice. Given this context, the present study aimed to characterize the pathotypes and the antimicrobial resistance profile of *E. coli* strains isolated from fecal samples of dogs and cats sent to a private laboratory.

## 2. Materials and Methods

### 2.1. Sample Processing and Bacteria Identification

Feces from 31 dogs and 3 cats were obtained from a commercial diagnostic laboratory located in Botucatu, Brazil, from March 2021 to October 2021.

The fecal samples were homogenized and, with a sterile swab, were streaked on Petri plates with MacConkey agar (Newprov, Pinhais, Brazil) and incubated at 37 °C for 24 h. Three lactose fermenting and morphologically compatible to *E. coli* colonies from each sample were selected, streaked on blood agar, and incubated at 37 °C for 24 h to confirm colony isolation. The colonies were stored in 1% Tryptic Soy Agar (BD, Sparks, NV, USA) at room temperature and in Tris-EDTA pH 7.4 (TE, 0.1 M Tris, 0.01 M EDTA) at −20 °C for further analysis. The isolated bacterial species were confirmed using the Matrix-Assisted Laser Desorption Ionization Time-of-Flight (MALDI-TOF MS) (Bruker, Billerica, MA, USA).

### 2.2. Antimicrobial Susceptibility Testing and Phenotypic Characterization of Extended-Spectrum Beta-Lactamase

The antimicrobial susceptibility profile was determined using the disk diffusion test (Kirby–Bauer method) and breakpoints according to the Clinical and Laboratory Standards Institute (CLSI) protocol [11] using the following drugs and concentrations (DME, Araçatuba, Brazil): cefoxitin 30 µg (CFO), ciprofloxacin 5 µg (CIP), gentamicin 10 µg (GEN), sulfamethoxazole 23.5 µg/trimethoprim 1.25 µg (SUT), amikacin 30 µg (AMI), ertapenem 10 µg (ERT), enrofloxacin 5 µg (ENO), and tetracycline 30 µg (TET).

The double-disk synergy test was performed to identify ESBL-producing *E. coli* [12] using amoxicillin–clavulanate 30 µg (AMC) and ceftriaxone 30 µg (CRO), ceftazidime 30 µg (CAZ), cefepime 30 µg (CPM), or aztreonam 30 µg (ATM). Plates were incubated at 35 °C for 18–20 h and the test was positive when the extension of the inhibition zone was observed between AMC and other cephalosporins.

### 2.3. DNA Extraction and Bacterial Genotypic Analysis

For bacterial DNA extraction, *E. coli* colonies were suspended in phosphate-buffered saline (pH 7.4), incubated at 99 °C for 10 min, cooled at –20 °C for 5 min, and centrifuged at 16,000 rpm for 10 min [13]. The supernatant was collected and stored at −20 °C until biomolecular analysis took place.

*E. coli* strains were classified in phylogroups (A, B1, B2, C, D, E, and F) according to the Clermont protocol [14], which is based on the presence/absence of the following genes: *arpA*, *chuA*, *yjaA*, *trpA,* and TspE4.

The pathotypes were characterized based on the detection of *stx1*, *stx2*, *eae*, *aggR*, *cnf1,* and *cnf2* genes [5,15,16,17]. Then, the strains were classified according to the gene amplification: STEC (*stx*), EHEC (*stx* and *eae*), EPEC (*eae*), EAEC (*aggR*), and NTEC (*cnf*) [16]. *E. coli* strains DL933 (*eaeA*, *stx1*, *stx2*, *ehxA*, *iha*, *toxB*, and *efa1*), EAEC O42 (*astA*, *aggR*, *aaf*, and *pet*), and S5 (*cnf*) were used as positive controls.

Finally, the presence of the *bla*_CTX-M_ gene group was searched in all ESBL-positive strains in the phenotypic test, according to Bonnet et al. (2001) [18].

Antimicrobial resistance, phylogroups, and virulence-factor-encoding genes were searched by conventional PCR according to their respective references, followed by an electrophoresis reaction for 2 h in 2% agarose gel stained with SYBR Safe DNA Gel Stain (Thermo Fisher Scientific Inc., Carlsbad, CA, USA) and analyzed by photodocumentation under ultraviolet light at 800 nm. All primer sequences, melting temperatures, and amplicon sizes are described in Table 1.

### 2.4. Statistical Analysis

Data were analyzed using Microsoft Excel ed. 2020 (Microsoft, Redmond, WA, USA). The data were used for descriptive statistical analysis to determine the frequencies of *E. coli* phylogroups (A, B1, B2, C, D, E, and F), virulence factor genes (*eae*, *ehxA*, *stx1,* and *stx2*), antimicrobial susceptibility, and ESBL-producing *E. coli* isolates.

## 3. Results

A total of 97 *E. coli* isolates were obtained from fecal samples; 88 were isolated from dogs and 9 were isolated from cats. Most of the isolates belonged to phylogroup A (29.9%; 29/97), followed by phylogroup B2 (26.8%; 26/97), phylogroup F (19.6%; 19/97), phylogroup D (13.4%; 13/97), phylogroup B1 (4.1%; 4/97), phylogroup C (4.1%; 4/97), and phylogroup E (2.1%; 2/97) (Table 2). The *E. coli* isolates obtained from dogs had a similar phylogroup profile to the total amount. But the isolates from cats were mainly from phylogroup D (33.3%; 3/9), phylogroup B1 (33.3%; 3/9), phylogroup C (22.2%; 2/9), and phylogroup E (11.1; 1/9). Regarding the virulence components, *eae* gene was detected in 20 of all isolates, 19 isolates from dogs (21.6%; 19/88), and 1 isolate from a cat (11.1%; 1/9), and none of the components had the *stx1*, *stx2*, *aggR*, *cnf1*, or *cnf2* genes. Therefore, 20.6% of isolates (20/97) were classified as EPEC.

The antimicrobial susceptibility test revealed that SUT (25.8%; 25/97) and TET (27.8%; 27/97) were the antimicrobials that isolates were most often resistant to. Resistance to both drugs was observed in 16.5% (16/97) isolates (Table 3). Thirteen isolates (13.4%) were characterized as multidrug-resistant [21], and EPEC was resistant to SUT (10.0%; 2/20) and TET (20.0%; 4/20) only.

Finally, five isolates (5.1%; 5/97) were ESBL-producing and *bla*_CTX-M_-positive bacteria. Moreover, most ESBL-producing *E. coli* isolates (80%; 4/5) were phylogroup A, followed by phylogroup F (20%; 1/5).

## 4. Discussion

In the current study, EPEC and MDR *E. coli* strains, including ESBL-producing isolates, from fecal samples of dogs and cats were identified and characterized.

Regarding the prevalence of EPEC strains, our results surpass those of other comparable investigations, revealing higher prevalence rates compared to previous studies, such as 14.9% for dogs [22] and 11.8% for cats [23], which reported EPEC in fecal and rectal swab samples from these animals. Our study suggests that dogs and cats may shed EPEC differently. Consistent with the findings of Punõ-Sarmiento et al. (2013) [23], we observed that dogs are apparently carriers of EPEC more frequently than cats.

The isolation of EPEC in dog feces raises concerns about the potential role of companion animals as reservoirs for DEC and their involvement in the zoonotic transmission of these strains. Although the effects of these strains in dogs and cats are not fully understood, it is known that the intimin Eae protein can cause attaching and effacing lesions in the intestinal epithelium [2]. This damage impairs intestinal absorption and can result in acute diarrhea, with the effects being particularly severe in children [2].

Numerous factors contribute to the diversity of *E. coli* strains in the fecal microbiota of dogs and cats. As highlighted by Treier et al. (2021) [24], the interplay of feeding practices and the overall health status of these animals, as emphasized by Carvalho et al. (2021) [25], significantly influences the shedding of bacterial fecal matter. Regarding microbiota profiles, particularly the *E. coli* phylogroups and virulence factors, variations are observed between healthy and diarrheagenic dogs, as reported by Coura et al. (2018) [22]. In this study, the predominant *E. coli* phylogroups were A and B2, in contrast to many other studies that commonly report a high prevalence of B1 and A phylogroups, often associated with commensal strains [25,26,27,28]. Phylogroup B2 *E. coli*, on the other hand, is frequently linked to extraintestinal infections in canines [29,30]. Additionally, some studies highlight a high prevalence of B1 and D in fecal samples [28,29], suggesting that the variation in phylogroups may be multifactorial, influenced by factors such as region, contacts, feeding practices, and overall health status.

Antimicrobial resistance is a One Health challenge; therefore, it poses a transboundary threat against human, animal, and environmental health. It stems from the overuse and misuse of antimicrobials and also represents a menace at the socioeconomic level. Bacteria can exchange genetic material through horizontal gene transfer processes, such as conjugation, transformation, and/or transduction. When incorporating DNA from other bacteria, they may acquire antimicrobial-resistant genes, promoting the emergence of multidrug-resistant (MDR) strains. MDR bacteria, resistant to three or more classes of antimicrobials [21], hold significant clinical relevance, as high mortality rates are attributed to MDR infections, mainly caused by Gram-negative bacteria [31]. In this study, approximately 13% of *E. coli* were identified as MDR, a rate higher than that reported by Harada et al. (2012) [29] in dogs from Japan. Furthermore, our data agree with other reports that described MDR belonging mainly to phylogroups C and A [25,27,32], whereas Sato et al. (2014) [33] reported more MDR *E. coli* from dogs belonging to phylogroup D.

ESBL-producing *E. coli* were isolated from dog samples and, similarly to other studies, isolated ESBL-producing bacteria were prevalent in phylogroup A [25,26,27]. Also, the primer to detect *bla*_CTX-M_ was originally designed to detect CTX-M-1-, CTX-M-2-, and CTX-M-9-like encoding genes [18], and perhaps other variants could not be detected.

ESBL are enzymes produced by specific bacterial strains that can destroy first-, second-, and third-generation cephalosporin molecules, such as ceftazidime, ceftriaxone, cefotaxime, and penicillin and aztreonam (a monobactam) [34]. Due to the therapy restriction, ESBL infections pose a serious threat to life. The most critical ESBLs are part of TEM, SHV, CTX-M, and certain OXA enzyme classes, which are expressed by *bla* genes, the dominant class of antimicrobial-resistant genes [35]. The resistance to extended-spectrum cephalosporins in *E. coli* was frequently attributed to CTX-M variants, expressed by the *bla*_CTX-M_ gene, the most common and widespread ESBL gene in animals and humans [36,37]. In the present research, *bla*_CTX-M_ genes were identified in all ESBL-producing strains. In fact, *bla*_CTX-M_, especially *bla*_CTX-M-15_, is very disseminated in *E. coli* strains from dogs’ gut microbiota. Although the *bla*_CTX-M-15_ gene has been the most frequently ESBL-related gene detected in *E. coli* from dogs [25], other *bla*_CTX-M_ variants [25,26,27], in addition to other cephalosporinase-encoding genes such as *bla*_CMY-*2*_ [25,27] and *bla*_SHV-12_ [26], have also been reported in canine feces. These findings underscore the widespread prevalence of ESBL-producing *E. coli* in the intestinal environment of dogs, emphasizing the need for cautious handling. Even healthy animals can carry ESBL-producing bacteria, posing a risk of transmitting these microbes to the environment and thereby endangering humans and other animals with the potential for challenging-to-treat infections.

The use of samples obtained from a private microbiology veterinary laboratory highlights the potential of these facilities for the surveillance of antimicrobial-resistant bacteria. There are some national collaborative systems that integrate private laboratories and public health systems aiming to put under surveillance antimicrobial-resistant bacteria. The most well structured are the CDC’s Antimicrobial Resistance Laboratory Network (AR Lab Network) [38] and the Spanish Network of Laboratories for the Surveillance of Resistant Microorganisms (RedLabRA) [39], which provide molecular epidemiologic analysis from obtained MDR strains.

Considering the Brazilian public health system and the fact that South American countries have concerning previsions about MDR pathogens [40], a similar collaborative system with public health agencies and human and veterinary microbiology laboratory facilities could be implemented.

## 5. Conclusions

In this study, a diversity in *E. coli* phylogroups was observed, along with a high prevalence of enteropathogenic *E. coli* strains, as well as the presence of multidrug-resistant isolates in fecal samples from both dogs and cats.

As limitations, we cite the lack of information regarding the health status of the animals; such information would be valuable to correlate EPEC isolation to possible clinical signs in those pets. The relatively low frequency of samples from cats also underscores a lack of information about the *E. coli* profile in the feline microbiota. Therefore, it is relevant to investigate pathogenic *E. coli* strains and antimicrobial resistance profiles in cats, particularly given the popularity of this species as pets. Also, the processing of stool samples in private laboratories is intensive, primarily focusing on the detection of parasites such as intestinal worms, but also for bacteria. In this context, laboratories should actively contribute to antimicrobial resistance tracking by routinely screening for fecal pathogenic MDR bacteria. This ongoing surveillance is crucial for partner hospitals and clinics to gain insights into the bacteria circulating in the community. It enables the development of biosecurity measures and guidelines for antimicrobial use, ultimately aiding in the prevention of antimicrobial resistance dissemination.

## Figures and Tables

**Table 1:** Primer sequences and amplicon size for detection of antimicrobial resistance, phylogroup identification, and virulence genes.

Function-Related Group	Target Gene	Primer Sequences	Amplicon Size (bp)	Reference
Antimicrobial resistance	*bla*_CTX-M_	5′-CGCTTTGCGATGTGCAG-3′	550	[18]
		5′-ACCCGCGATATCGTTGGT-3′		
Phylogroup	*chuA*	5′-ATGGTACCGGACGAACCAAC-3′	288	[14]
		5′-TGCCGCCAGTACCAAAGACA-3′		
	*yjaA*	5′-CAAACGTGAAGTGTCAGGAG-3′	211	[14]
		5′-AATGCGTTCCTCAACCTGTG-3′		
	TspE4.C2	5′-CACTATTCGTAAGGTCATCC-3′	152	[14]
		5′-AGTTTATCGCTGCGGGTCGC-3′		
	*arpA*	5′-AACGCTATTCGCCAGCTTGC-3′	400	[14]
		5′-TCTCCCCATACCGTACGCTA-3′		[14,19]
	*arpA*	5′-GATTCCATCTTGTCAAAATATGCC-3′	301	[20]
		5′-GAAAAGAAAAAGAATTCCCAAGAG-3′		
	*trpA*	5′-AGTTTTATGCCCAGTGCGAG-3′	219	[20]
		5′-TCTGCGCCGGTCACGCCC-3′		
Virulence factors	*stx1*	5′-TTCGCTCTGCAATAGGTA-3′	555	[15]
		5′-TTCCCCAGTTCAATGTAAGAT-3′		
	*stx2*	5′-GTGCCTGTTACTGGGTTTTTCTTC-3′	118	[15]
		5′-AGGGGTCGATATCTCTGTCC-3′		
	*eae*	5′-ATATCCGTTTTAATGGCTATCT-3′	425	[15]
		5′-AATCTTCTGCGTACTGTGTTCA-3′		
	*aggR*	5′-CAGAATACATCAGTACACTG-3′	433	[16]
		5′-GAAGCTTACAGCCGATATA-3′		
	*cnf1*	5′-GAACTTATTAAGGATAGT-3′	543	[17]
		5′-CATTATTTATAACGCTG-3′		
	*cnf2*	5′-AATCTAATTAAAGAGAGAAC-3′ 5′-CATGCTTTGTATATCTA-3′	543	[17]

**Table 2:** Frequency of virulence genes and antimicrobial resistance profile of *E. coli* for each phylogroup.

Phylogroup		Antimicrobial Resistance Profile% (N)										Virulence Profile% (N)
	Total	ESBL	MDR	CFO	CIP	GEN	SUT	AMI	ERT	ENO	TET	EPEC *(eae)*
A	29	13.8 (4)	24.1 (7)	0.0	17.24 (5)	10.3 (3)	31.0 (9)	3.5 (1)	0.0 (0)	10.3 (3)	20.7 (6)	24.1 (7)
B1	4	0.0	0.0	0.0	0.0	0.0	0.0	0.0	0.0	0.0	0.0	0.0
B2	26	0.0	7.7 (2)	3.9 (1)	0.0	7.7 (2)	26.9 (7)	7.7 (2)	7.7 (2)	0.0	34.6 (9)	19.2 (5)
C	4	0.0	50.0 (2)	0.0	50.0 (2)	0.0	50.0 (2)	0.0	0.0	50.0 (2)	50.0 (2)	25.0 (1)
D	13	0.0	7.7 (1)	0.0	0.0	7.7 (1)	46.2 (6)	7.7 (1)	7.7 (1)	7.7 (1)	61.5 (8)	7.7 (1)
E	2	0.0	0.0	0.0	0.0	0.0	0.0	0.0	0.0	0.0	0.0	100.0 (2)
F	19	5.3 (1)	5.3 (1)	0.0	5.3 (1)	5.3 (1)	5.3 (1)	0.0	0.0	5.3 (1)	10.5 (2)	21.0 (4)
Total (97)		5.2 (5)	13.4 (13)	1.0 (1)	8.2 (8)	7.2 (7)	25.8 (25)	4.1 (4)	3.1 (3)	7.2 (7)	27.8 (27)	20.6 (20)

Extended-spectrum beta-lactamase (ESBL); multidrug-resistant (MDR); cefoxitin (CFO); ciprofloxacin (CIP); gentamicin (GEN); sulfamethoxazole + trimethoprim (SUT); amikacin (AMI); ertapenem (ERT); enrofloxacin (ENO); tetracycline (TET); enteropathogenic *E. coli* (EPEC).

**Table 3:** Frequency of each antimicrobial resistance profile in *E. coli* isolates.

Antimicrobial Resistance Profile	*E. coli* Isolates	
AMI	1.0%	(1/97)
CIP	1.0%	(1/97)
ENO	1.0%	(1/97)
SUT	5.2%	(5/97)
TET	9.3%	(9/97)
CIP-SUT	1.0%	(1/97)
SUT-TET	10.3%	(10/97)
AMI-ERT-TET	1.0%	(1/97)
CIP-ENO-GEN-SUT	2.1%	(2/97)
AMI-CFO-ERT-SUT-TET	1.0%	(1/97)
AMI-ERT-GEN-SUT-TET	1.0%	(1/97)
CIP-ENO-GEN-SUT-TET	4.1%	(4/97)

Amikacin (AMI); ciprofloxacin (CIP); enrofloxacin (ENO); sulfamethoxazole + trimethoprim (SUT); tetracycline (TET); gentamicin (GEN); ertapenem (ERT); cefoxitin (CFO).

## Data Availability

The original contributions presented in the study are included in the article, further inquiries can be directed to the corresponding author.
